# Supplementary material for: Sea lice (Lepeophtherius salmonis) detection and quantification around aquaculture installations using environmental DNA
Source: PLoS One. 2022 Sep 21;17(9):e0274736. doi: 10.1371/journal.pone.0274736 (PMC9491551; doi:10.1371/journal.pone.0274736)
Supplement: S1 Appendix — (DOCX) [file pone.0274736.s001.docx]

**S1 Appendix. Evaluation of the content of amplicons generated using SL2 assay**

The amplicons were generated firstly by pooling 72 qPCR-based amplicons (amplicons of qPCR reactions performed for 36 field samples collected in May 2020) and secondly by reamplification using the qPCR *L. salmonis* SL2 primer set (forward: 5′- ACATCGAGGTCACGAATATCTTT -3′ and reverse: 5′- ACCTTGTTTGGCTGGGATAG -3′). A single 100 µL PCR reaction consisted of 50 µL AmpliTaq Gold®360 PCR Master Mix (Thermo Fisher Scientific, Carlsbad, California, USA), 10 µL enhancer, 2.5 µL template DNA, distilled water, and each primer to final a concentration of 250 nM. PCR reaction cycling conditions were as follows: 95 °C for 10 min, followed by 25 cycles of 95 °C for 30 s, 58 °C for 40 s, 72 °C for 30 s, with a final extension step at 72 °C for 5 min. Three amplicons were sequenced on MiSeq Illumina sequencer. The raw sequencing data were processed using USEARCH v10.0.240_i86linux32 according to recommendations by Robert Edgar (1, 2). The sequences were merged and filtered using a Q20 quality threshold and length threshold of maximum 100 bp. If sequencing reads did not meet the quality requirements they were discarded. Following this, unique sequences were extracted from the merged reads, OTU clustering (at 97% similarity) and a de novo chimera identification step were performed using the UPARSEOTU algorithm. In the final step individual OTUs sequences were blasted against the *L. salmonis* mitochondrial reference sequence from GenBank (ID EU288200) using BLASTN 2.5.0+ (NCBI).

**Nucleotide sequence accession numbers:** Sequences obtained by Illuminia sequencing were deposited under the following GeneBank NCBI BioSample accession number: SAMN29936430

**References**

1. Edgar RC. Search and clustering orders of magnitude faster than BLAST. Bioinformatics. 2010;26(19):2460-1.

2. Edgar RC, Haas BJ, Clemente JC, Quince C, Knight R. UCHIME improves sensitivity and speed of chimera detection. Bioinformatics (Oxford, England). 2011;27(16):2194-200.
