# Supplementary figures and images for: Sea lice (Lepeophtherius salmonis) detection and quantification around aquaculture installations using environmental DNA
Source: PLoS One. 2022 Sep 21;17(9):e0274736. doi: 10.1371/journal.pone.0274736 (PMC9491551; doi:10.1371/journal.pone.0274736)

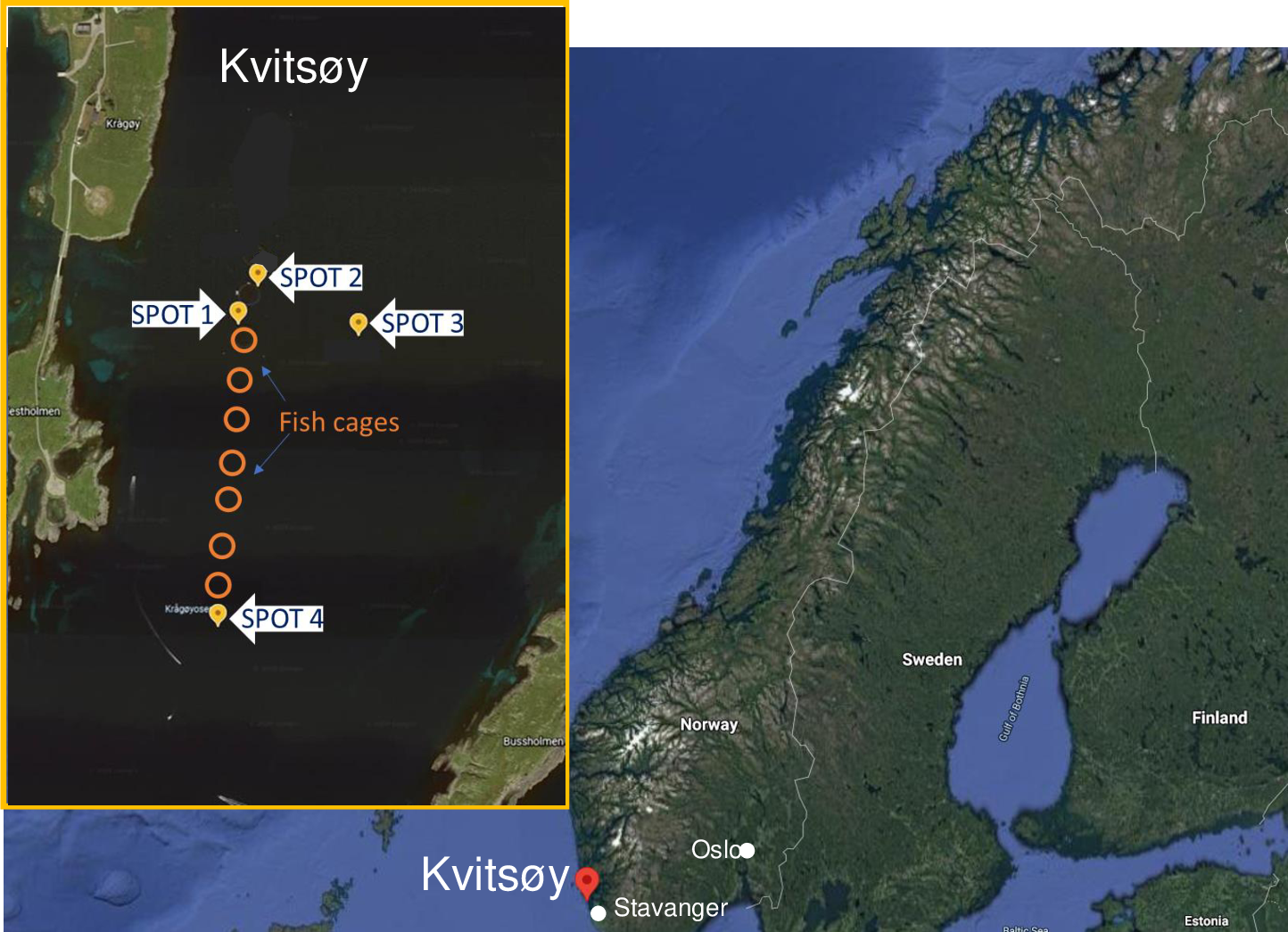

Supplement: S1 Fig — (TIF) [file pone.0274736.s002.tif]

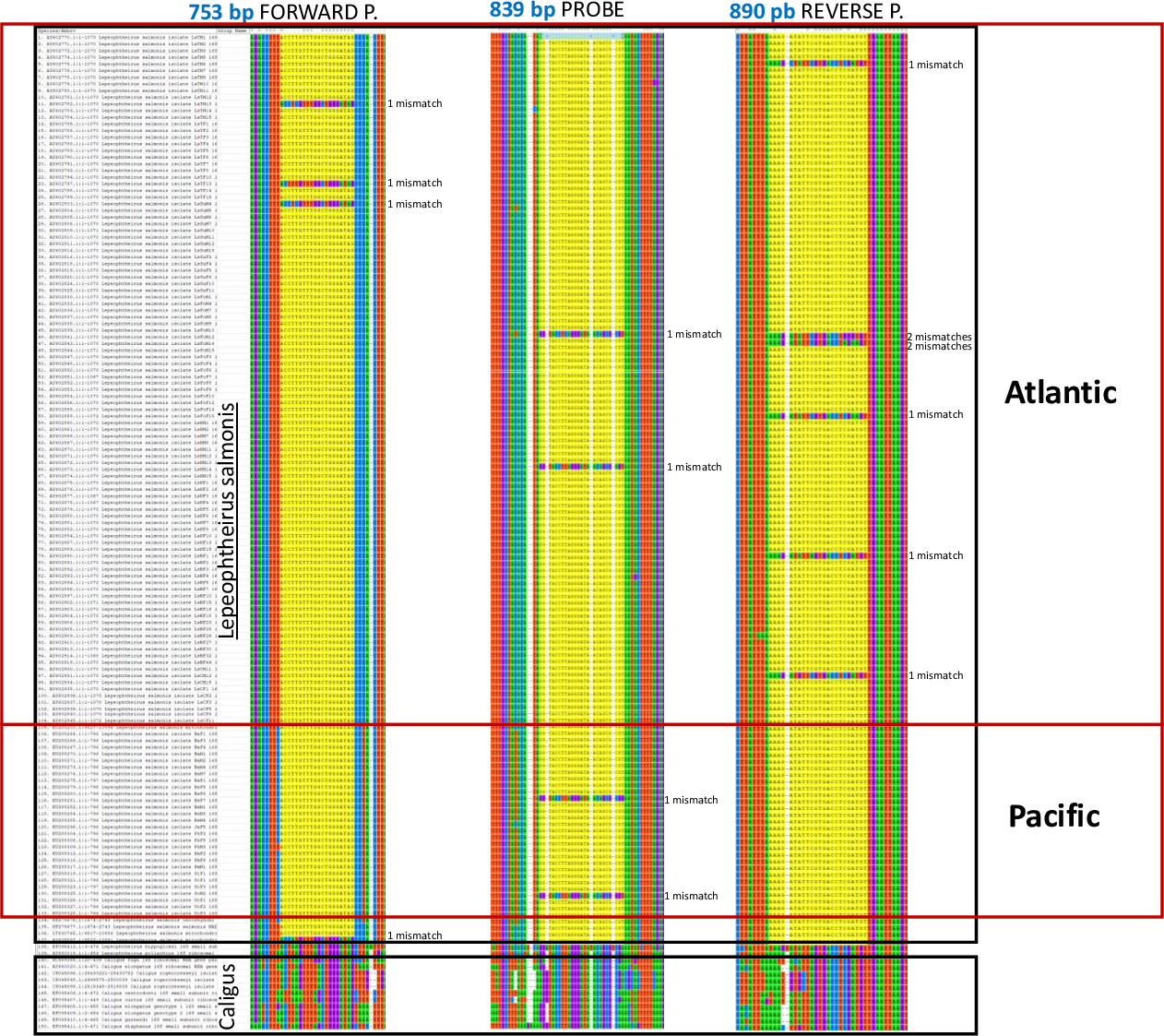

Supplement: S2 Fig — Fragments of the alignment generated for the randomly picked 136 (from 256) L. salmonids mitochondrial 16S rRNA sequences with regions primers and probe of qPCR assay target (on yellow). The number on blue–the start position (including gaps) for the oligo binding. In addition, for L. salmonids number of mismatches are provided. (TIF) [file pone.0274736.s003.tif]

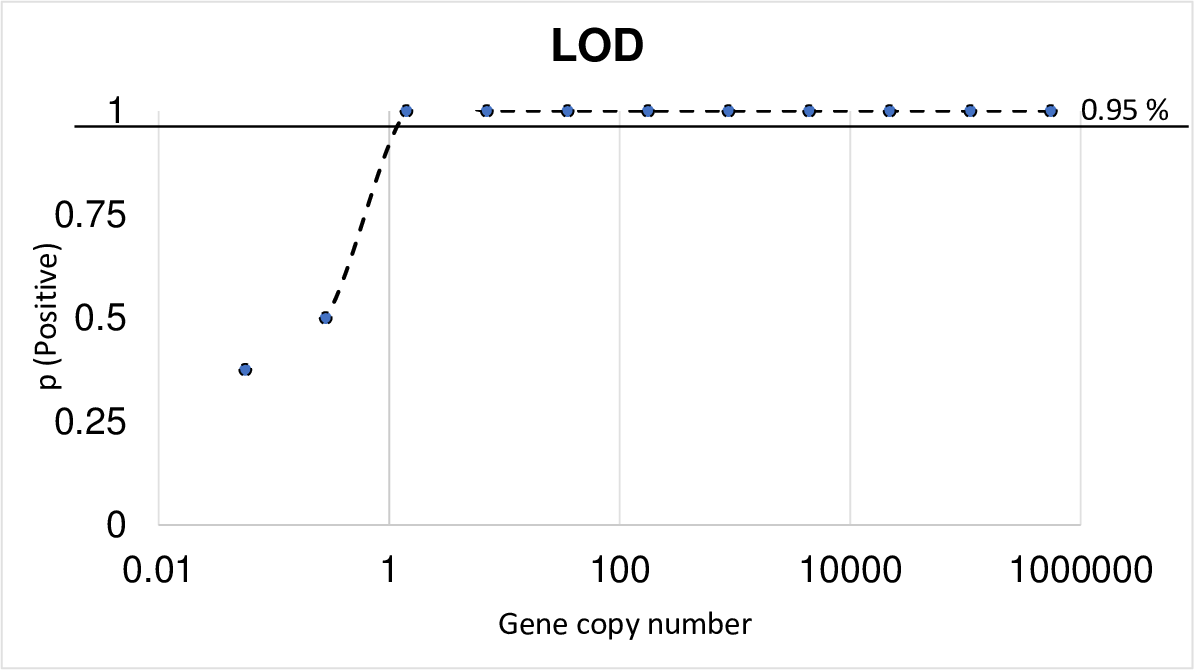

Supplement: S3 Fig — LOD was determined from dilution series, 8 replicates were amplified at concentration of 550000, 110000, 22000, 4400, 880, 176, 35.2, 7.04, 1.408, 0.2816, 0.05632 and O copies 1μl-1. The proportion of positive amplifications are plotted against the standard concentrations (x- axis logarithmic). LOD was determined as the minimum concentration of 95% replicates amplified (95% threshold is shown as a line). (TIF) [file pone.0274736.s004.tif]

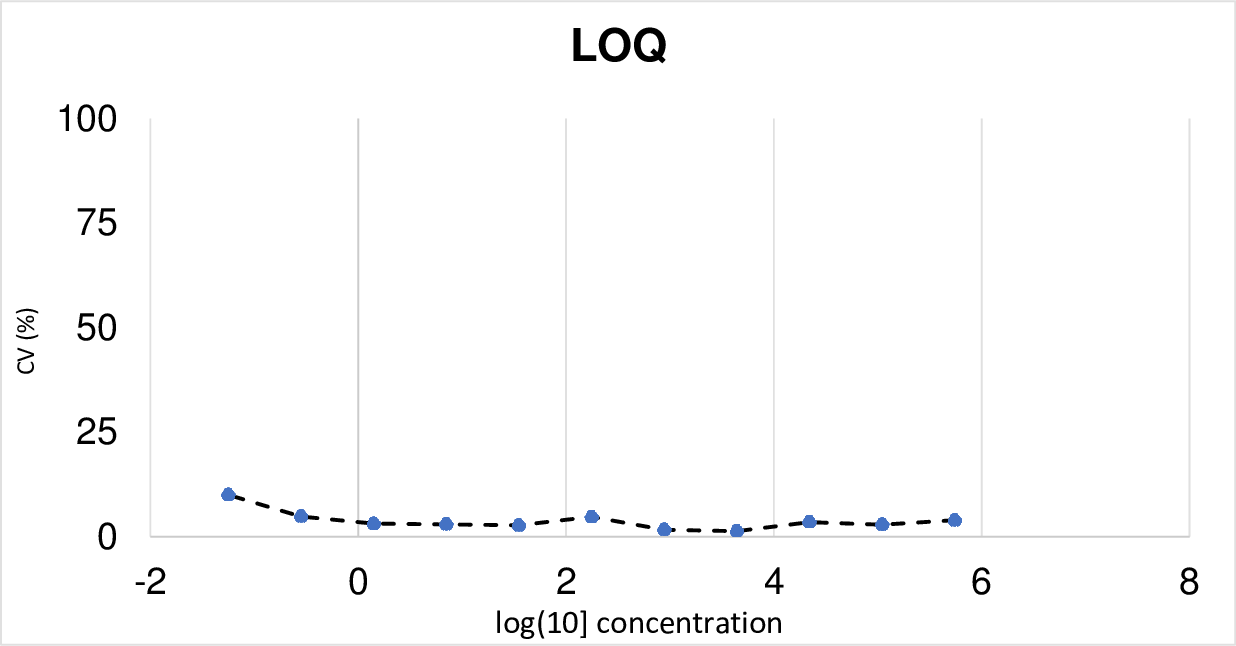

Supplement: S4 Fig — LOQ was determined from dilution series, 8 replicates were amplified at concentration of 550000, 110000, 22000, 4400, 880, 176, 35.2, 7.04, 1.408, 0.2816, 0.05632 1μl-1. The coefficient of variation (relative standard deviation) (CV = 100*(SD/mean)) was plotted against logarithmic transformed concentrations. (TIF) [file pone.0274736.s005.tif]

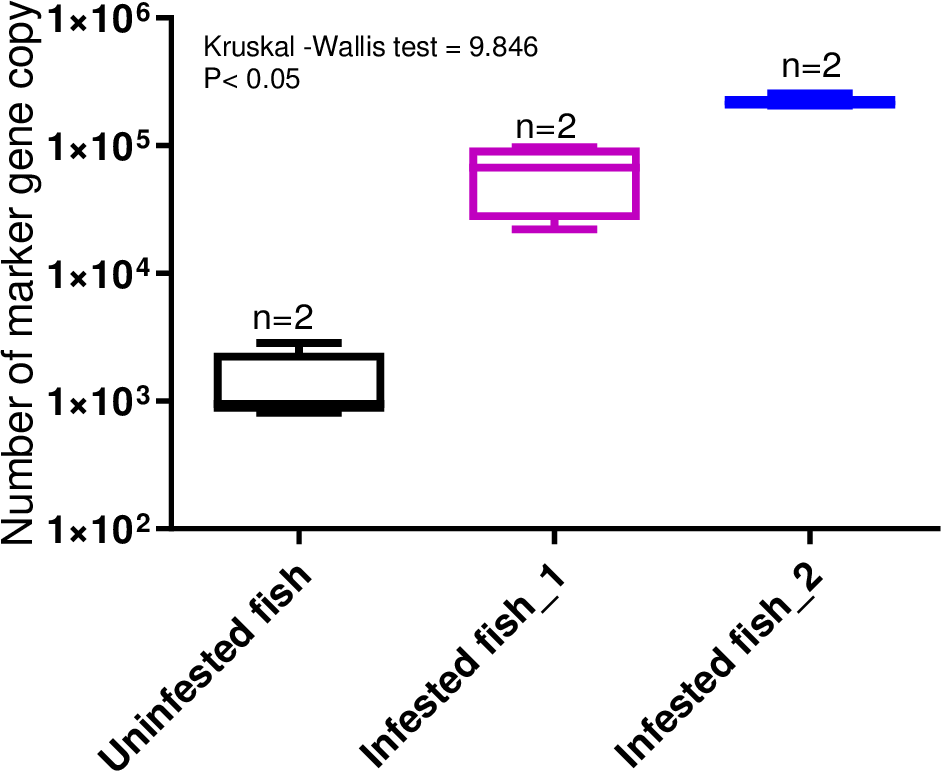

Supplement: S5 Fig — (TIF) [file pone.0274736.s006.tif]

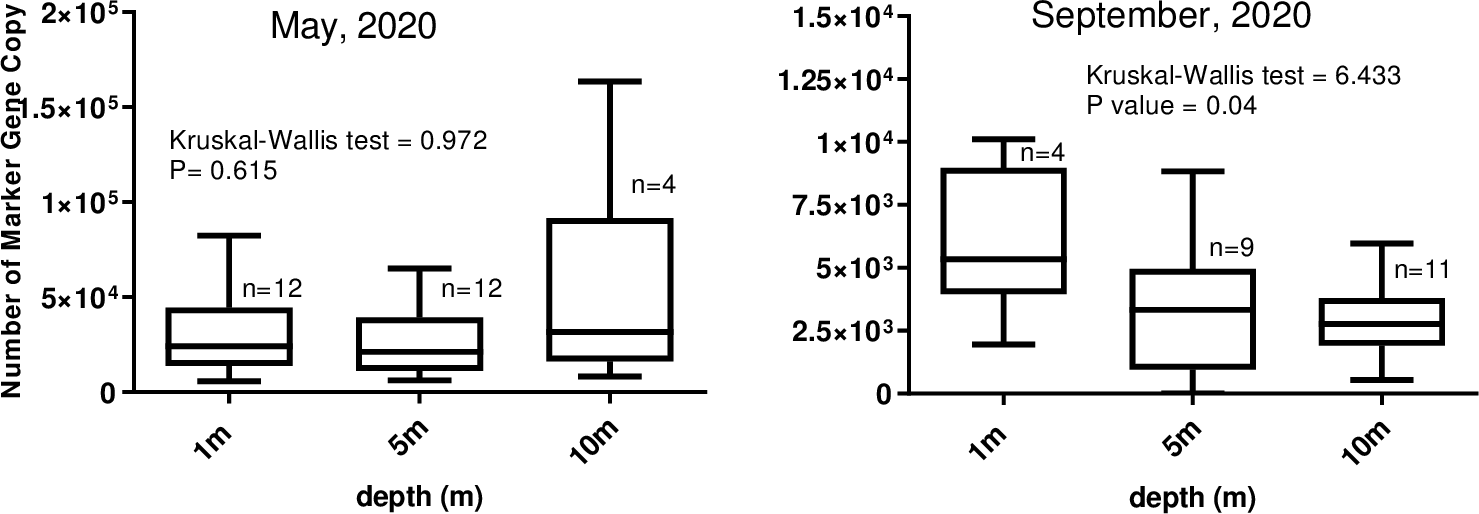

Supplement: S6 Fig — N is number of samples included. (TIF) [file pone.0274736.s007.tif]

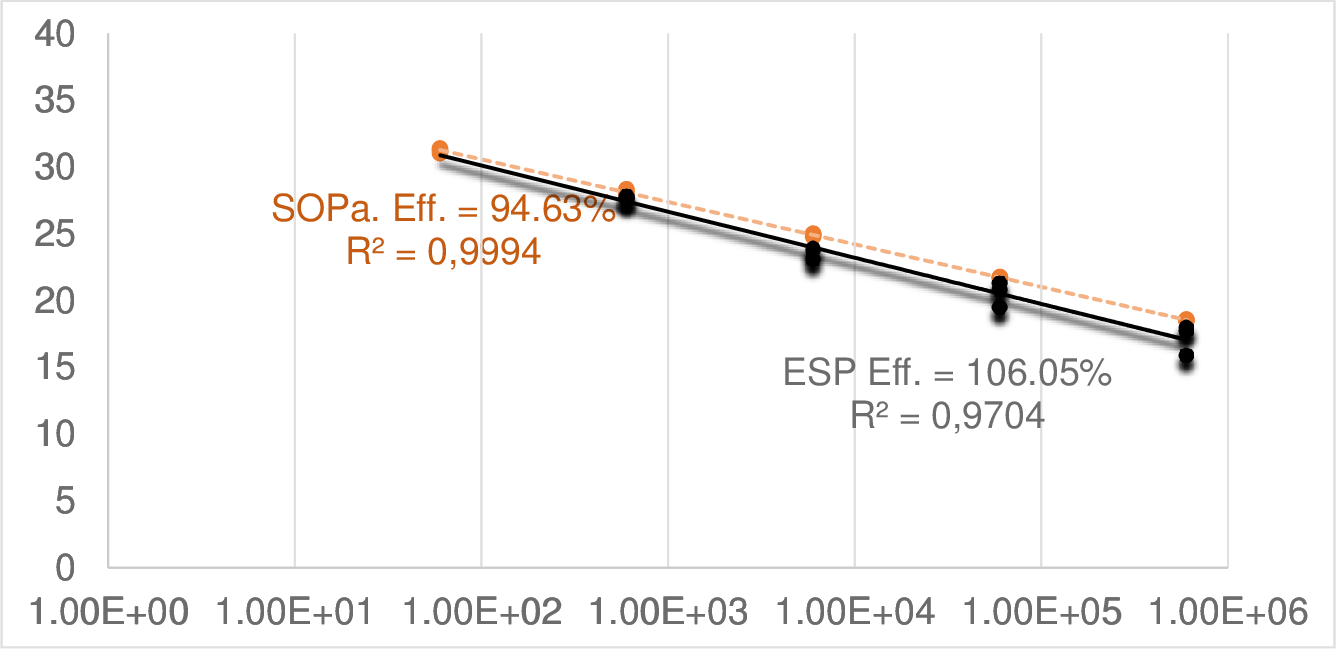

Supplement: S7 Fig — Y-axis -Ct value, X-axis -gene copy number per 1 μL. (TIF) [file pone.0274736.s008.tif]

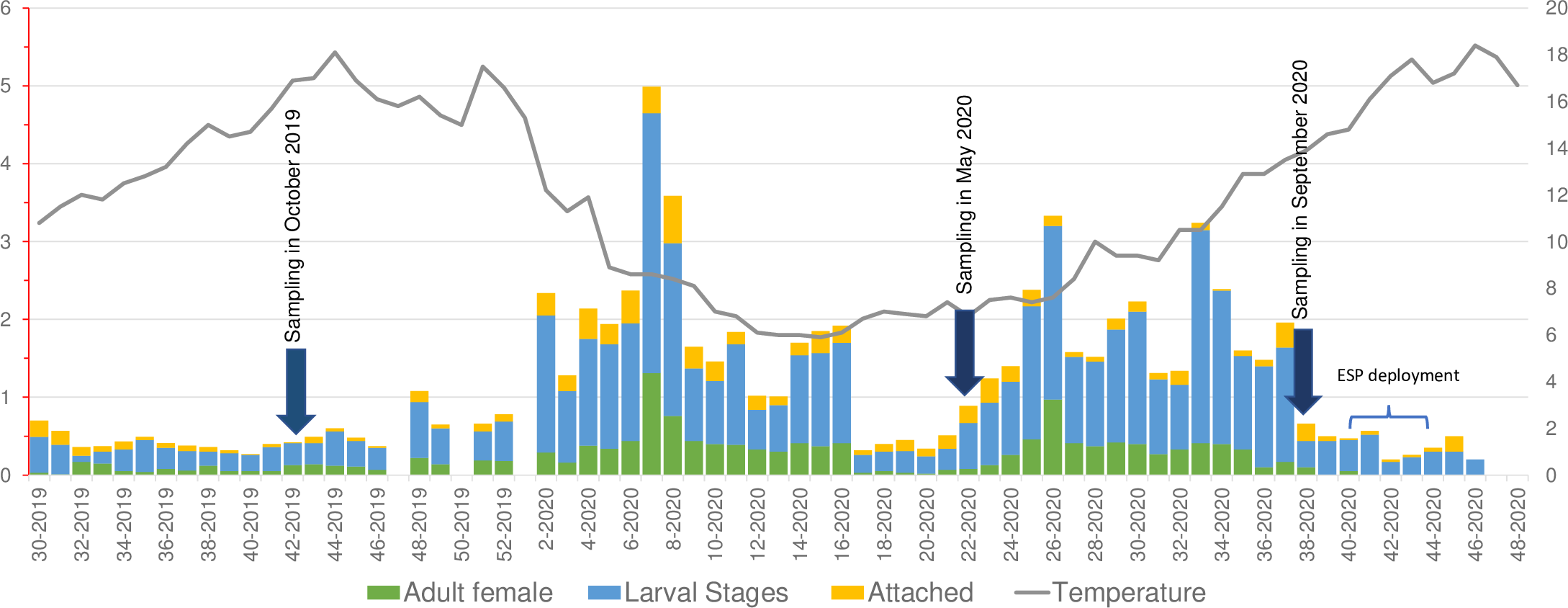

Supplement: S8 Fig — (TIF) [file pone.0274736.s009.tif]
