# Supplementary material for: Sea lice (Lepeophtherius salmonis) detection and quantification around aquaculture installations using environmental DNA
Source: PLoS One. 2022 Sep 21;17(9):e0274736. doi: 10.1371/journal.pone.0274736 (PMC9491551; doi:10.1371/journal.pone.0274736)
Supplement: S1 Table — (PPTX) [file pone.0274736.s010.pptx]

## Slide 1
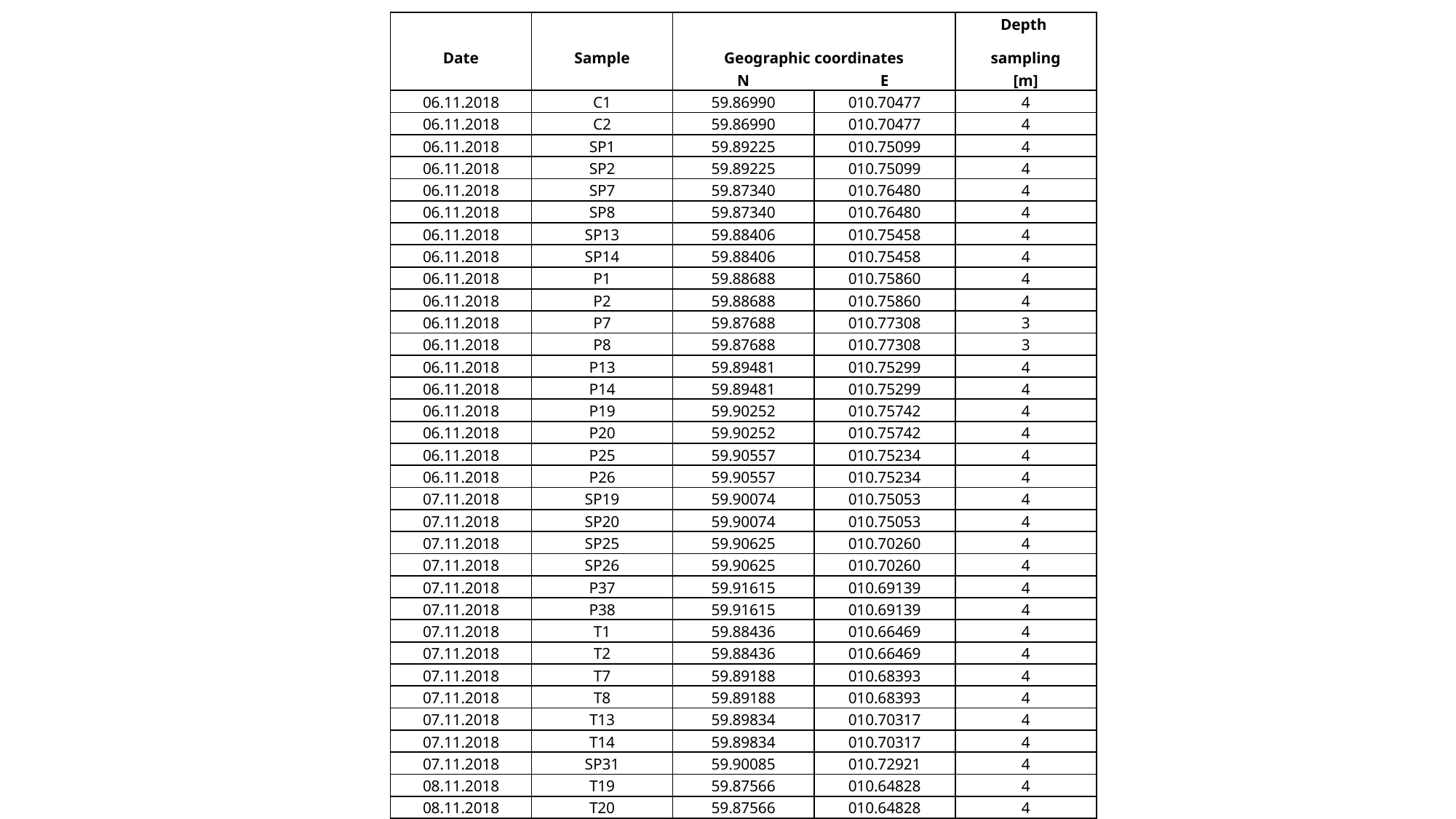

| Date | Sample | Geographic coordinates | | Depth sampling |
| --- | --- | --- | --- | --- |
| | | N | E | [m] |
| 06.11.2018 | C1 | 59.86990 | 010.70477 | 4 |
| 06.11.2018 | C2 | 59.86990 | 010.70477 | 4 |
| 06.11.2018 | SP1 | 59.89225 | 010.75099 | 4 |
| 06.11.2018 | SP2 | 59.89225 | 010.75099 | 4 |
| 06.11.2018 | SP7 | 59.87340 | 010.76480 | 4 |
| 06.11.2018 | SP8 | 59.87340 | 010.76480 | 4 |
| 06.11.2018 | SP13 | 59.88406 | 010.75458 | 4 |
| 06.11.2018 | SP14 | 59.88406 | 010.75458 | 4 |
| 06.11.2018 | P1 | 59.88688 | 010.75860 | 4 |
| 06.11.2018 | P2 | 59.88688 | 010.75860 | 4 |
| 06.11.2018 | P7 | 59.87688 | 010.77308 | 3 |
| 06.11.2018 | P8 | 59.87688 | 010.77308 | 3 |
| 06.11.2018 | P13 | 59.89481 | 010.75299 | 4 |
| 06.11.2018 | P14 | 59.89481 | 010.75299 | 4 |
| 06.11.2018 | P19 | 59.90252 | 010.75742 | 4 |
| 06.11.2018 | P20 | 59.90252 | 010.75742 | 4 |
| 06.11.2018 | P25 | 59.90557 | 010.75234 | 4 |
| 06.11.2018 | P26 | 59.90557 | 010.75234 | 4 |
| 07.11.2018 | SP19 | 59.90074 | 010.75053 | 4 |
| 07.11.2018 | SP20 | 59.90074 | 010.75053 | 4 |
| 07.11.2018 | SP25 | 59.90625 | 010.70260 | 4 |
| 07.11.2018 | SP26 | 59.90625 | 010.70260 | 4 |
| 07.11.2018 | P37 | 59.91615 | 010.69139 | 4 |
| 07.11.2018 | P38 | 59.91615 | 010.69139 | 4 |
| 07.11.2018 | T1 | 59.88436 | 010.66469 | 4 |
| 07.11.2018 | T2 | 59.88436 | 010.66469 | 4 |
| 07.11.2018 | T7 | 59.89188 | 010.68393 | 4 |
| 07.11.2018 | T8 | 59.89188 | 010.68393 | 4 |
| 07.11.2018 | T13 | 59.89834 | 010.70317 | 4 |
| 07.11.2018 | T14 | 59.89834 | 010.70317 | 4 |
| 07.11.2018 | SP31 | 59.90085 | 010.72921 | 4 |
| 08.11.2018 | T19 | 59.87566 | 010.64828 | 4 |
| 08.11.2018 | T20 | 59.87566 | 010.64828 | 4 |
| 08.11.2018 | T25 | 59.85912 | 010.62574 | 4 |
| 08.11.2018 | T26 | 59.85912 | 010.62574 | 4 |
| 08.11.2018 | SP37 | 59.89819 | 010.65137 | 4 |
| 08.11.2018 | SP38 | 59.89819 | 010.65137 | 4 |
| 08.11.2018 | P46 | 59.91871 | 010.67362 | 3,5 |
| 08.11.2018 | P47 | 59.91871 | 010.67362 | 3,5 |
