# Supplementary material for: Sea lice (Lepeophtherius salmonis) detection and quantification around aquaculture installations using environmental DNA
Source: PLoS One. 2022 Sep 21;17(9):e0274736. doi: 10.1371/journal.pone.0274736 (PMC9491551; doi:10.1371/journal.pone.0274736)
Supplement: S2 Table — (PPTX) [file pone.0274736.s011.pptx]

## Slide 1
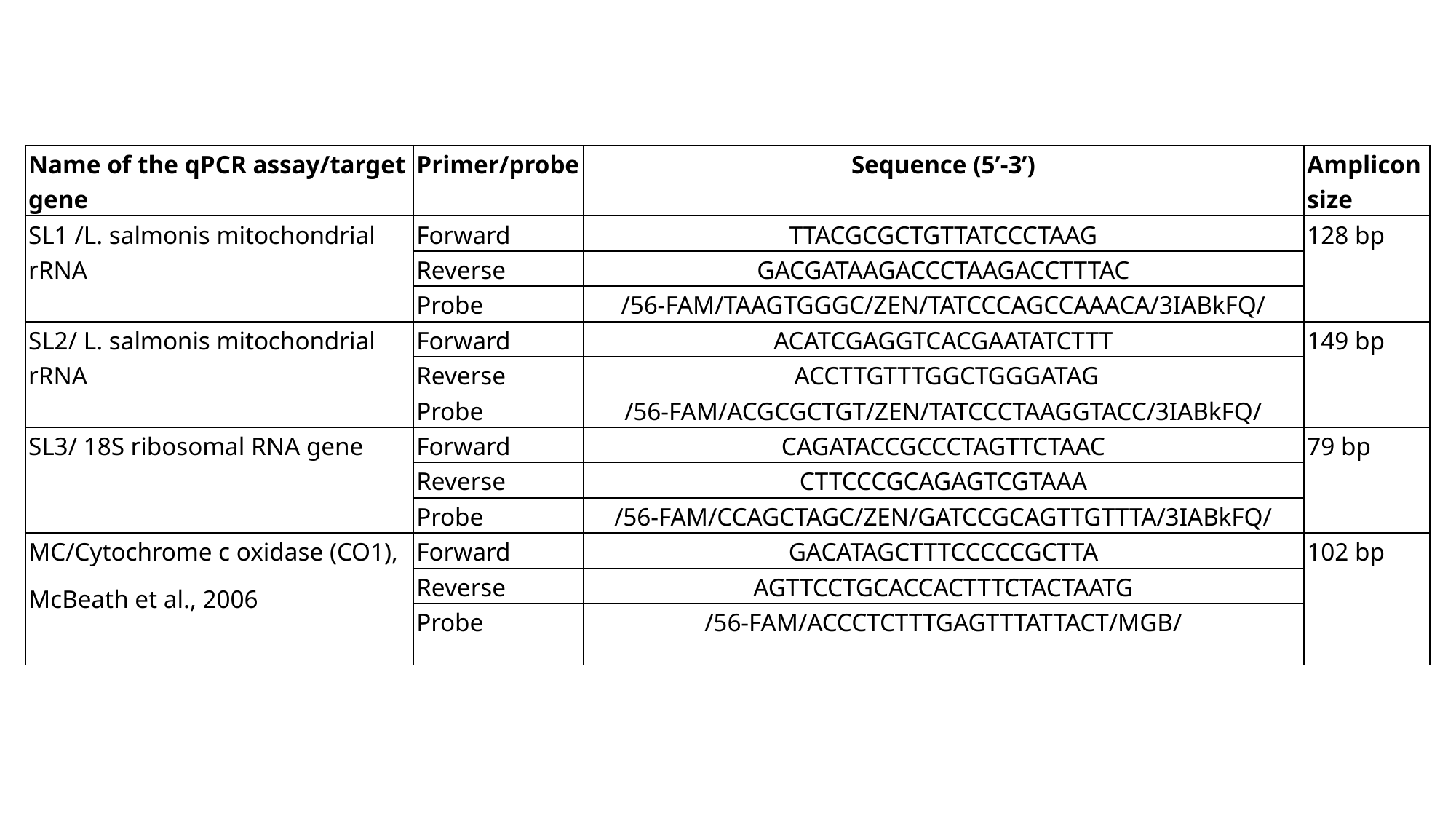

| Name of the qPCR assay/target gene | Primer/probe | Sequence (5’-3’) | Amplicon size |
| --- | --- | --- | --- |
| SL1 /L. salmonis mitochondrial rRNA | Forward | TTACGCGCTGTTATCCCTAAG | 128 bp |
| | Reverse | GACGATAAGACCCTAAGACCTTTAC | |
| | Probe | /56-FAM/TAAGTGGGC/ZEN/TATCCCAGCCAAACA/3IABkFQ/ | |
| SL2/ L. salmonis mitochondrial rRNA | Forward | ACATCGAGGTCACGAATATCTTT | 149 bp |
| | Reverse | ACCTTGTTTGGCTGGGATAG | |
| | Probe | /56-FAM/ACGCGCTGT/ZEN/TATCCCTAAGGTACC/3IABkFQ/ | |
| SL3/ 18S ribosomal RNA gene | Forward | CAGATACCGCCCTAGTTCTAAC | 79 bp |
| | Reverse | CTTCCCGCAGAGTCGTAAA | |
| | Probe | /56-FAM/CCAGCTAGC/ZEN/GATCCGCAGTTGTTTA/3IABkFQ/ | |
| MC/Cytochrome c oxidase (CO1), McBeath et al., 2006 | Forward | GACATAGCTTTCCCCCGCTTA | 102 bp |
| | Reverse | AGTTCCTGCACCACTTTCTACTAATG | |
| | Probe | /56-FAM/ACCCTCTTTGAGTTTATTACT/MGB/ | |
