# Supplementary material for: Sea lice (Lepeophtherius salmonis) detection and quantification around aquaculture installations using environmental DNA
Source: PLoS One. 2022 Sep 21;17(9):e0274736. doi: 10.1371/journal.pone.0274736 (PMC9491551; doi:10.1371/journal.pone.0274736)
Supplement: Attachment — Submitted filename: Rebuttal letter_1008.docx [file pone.0274736.s012.docx]

# **Rebuttal letter**

## 1. **Responses to the reviewers**

We would like to thank both reviewers for the valuable comments. The comments raised by the reviewers brought our attention to some of the important but less visible aspects of the manuscript regarding methodology, eDNA based detection and quantification of sea lice. Below our answers (on blue) to each point raised by the reviewers:

Reviewer #1: Krolicka et al. have developed a new assay to specifically detect and quantify sea lice eDNA. The manuscript is well written.
There are few minor points.
Line 43: control aquaculture related diseases - has been corrected
Line 82: Italicize “L. salmonis” -has been corrected
Line 178-185: Can you explain why the L. salmonis naupli or copepodites were directly spiked to the filter rather than spiking them to seawater and conducting filtration afterwords.

We consider this comment being highly justified. The decision of spiking directly to the filter has been made because the overarching goal was to establish possible relationship between an exact number of individuals for two different life-stages and gene copy number/quantification. Spiking seawater with sea lice would be source of possible variation and uncertainties, as the small larvae tend to be retained on the walls of the glass bottles and not be filtered, with the risk that the relationship between the sea lice life-stages in seawater and the eDNA quantification could be biased. In the field, we are aware that eDNA may arise also from free DNA in addition to bulk individuals, but the intention was here to obtain an empirical relationship between number of individual and the measured gene copy numbers and ignoring the potential contribution of free DNA.

Figure 1: Legends are too small -has been corrected
Line 332: “The numbers of MGC…” sentence may be not necessary. -the sentence has been deleted
Line 336: as 136 -has been corrected
Line 369: production limiting factors -has been corrected
Line 408: to some extent -has been corrected
Line 429-431: The idea of this sentence – the sentence has been modified
Line 467-473: Have you evaluated the temporal variation of L. salmonis eDNA?
Supplementary Figure 2: Pacific- has been corrected
Supplementary Figure 3: ….and 0 copies per µl -has been corrected
Supplementary Figure 5 and 6: Please show the statistical significance – this has been done

Reviewer #2: The author presents a novel and important validation for an eDNA-based approach to sea lice monitoring. Given the ongoing issue of sea lice infestation on Norwegian aquaculture sites and the elusive nature of unattached infective stages, this may provide a powerful tool for early detection of present/future infestation pressure. Overall, this is an interesting and comprehensive study addressing a question of great ecological and economic importance in Norway. I outline a few specific points below that I hope the author will try to address if relevant to the interpretation of their results.

In the case of samples collected from aquaculture sites, is it possible to discriminate between eDNA from nauplii/copepodite stages and eDNA shed from attached or dead adult/sub-adult lice? Given the potentially large contribution of DNA from these larger lice stages, the presence of high numbers of adult/sub-adult lice may obscure the interpretation of the abundance of infective stages. It may be valuable to discuss briefly the potential contribution of adult/sub-adult DNA from farm samples if the author can estimate from their collected data if they believe this may be relevant to the interpretation of their results.
The reviewer is touching upon a tricky aspect, which is general for the environmental DNA methodology. With that approach, we cannot reliably discriminate nauplii/copepodite stages from attached or dead adult/sub-adult lice. Basically, we have too little knowledge and data to discuss in the manuscript when it comes to “*the potentially large contribution of DNA from these larger lice stages, the presence of high numbers of adult/sub-adult lice may obscure the interpretation of the abundance of infective stages*”. Although we believe that there will not be a large disproportion if comes to these numbers because as the reviewer mentioned eDNA from the attached stages will be mainly the fraction of shed eDNA, meanwhile free-living stages will be found in water as whole organisms or as their tissue fragments. This means we may in some instances overestimate the gene copy numbers for free living stages i.e. **potential** for the high infestation pressure. eRNA based detection could overcome the issue of detection and quantification of dead/living organisms and possibly the fraction of larval/adult eDNA. However, the sensor device Environmental Sampling Processor (ESP) with implemented PCR module used here is currently only able only to quantify DNA gene targets. In the manuscript we were trying to underline that long lasting eDNA based investigation through several seasons coupled with the data gained by fish farmers about numbers of sea lice counted on fish could provide information about fluctuations, possibly pattern, and **provide support for how the eDNA based data could be interpreted**.

Lines 46-47: The author outlines their rationale for focusing on L. salmonis, as this is the primary sea lice species impacting Norwegian salmon farms. I am wondering if the author suspects this method could be applied to Caligus spp. Do you think special considerations would be required to apply such a method to Caligus spp. due to their broader host range or differences in lifecycle? Or do you think a similar eDNA-based monitoring method could be developed for members of this sea lice genus using a similar approach to that outlined in this this study?
Yes, this method could be applied to *Caligus spp*. Although the current regulation focus is on *L. salmonis*, we believe it would be useful to use a combination of eDNA based monitoring of *L. salmonis* and *Caligus sp*. (using an assay solely targeting *Caligus sp*.). That would provide more comprehensive information about sea lice infestation and threat to fish farms. The results based on the parallel monitoring would distinguish these two, especially this could be valuable in the geographic localities with larger impact from Caligus sp.

Lines 63-65: As the author describes, L. salmonis exhibit non-parasitic stages. I was wondering if the author has thoughts on how one might incorporate such information into such an eDNA-based monitoring program and/or if it would be necessary for the effectiveness of this monitoring approach. For example, is it possible that eDNA from these non-parasitic stages could be detected from a nearby farm source but that did not necessarily translate to an elevated infection risk for the focal farm (given the latent period prior to these free-living stages becoming infective)? It sounds like the primary proposed application of this methodology is to assess increases in infestation pressure on fish farms, in which case the presence of non-infective stages would likely be related to the abundance of adult lice at a given site. If the author has considered if/how the presence of these non-infective stages may be incorporated into the application of an eDNA-based monitoring program, it may be worth briefly discussing.
The response to this comment was partly made to the reviewer’s first comment. Some additional, longer lasting study is needed to understand the seasonal fluctuations of sea lice eDNA detection and quantifications, and how this influences the real risk of infection. This was not part of the scope of the project of this research due to the limited financial resources. We agree this evaluation could lead to a better understanding how this type of data could be used by fish farmers to take actions and be prepared. We believe that for some period both type of investigations, traditional monitoring and eDNA based will need to be performed in parallel and then the eDNA based information could be utilized with good confidence to replace/support the conventional method. Lines 390-400: The authors discuss ecological explanations for observed differences in depth stratified eDNA detections between sampling seasons. It may be worth discussing potential explanations for this phenomenon related to eDNA dispersal and/or stability. For example, is it possible that depth-dependent eDNA mixing and/or degradation rates were more variable in September than in May, independent of sea lice distributions?

We agree with the reviewer, therefore we have added a short text (below) to the manuscript about phenomenon related to eDNA dispersal and/or stability. We should not exclude the possibility that depth-dependent eDNA mixing and/or degradation rates were more variable in September than in May and this phenomenon independent or **partially independent** of sea lice distributions.

**“**Since the spatial and temporal signal of eDNA is dependent heavily on the environment it is possible that vertical mixing of water masses and/or degradation rates was more important than sea lice distributions. Season, light conditions, and water temperature are among the most important factors that impact distribution and eDNA stability (Harrison et al., 2019). The light conditions were different in May than September, UV light intensity was stronger in May than in September, therefore it is possible for example that UV light impacted on faster eDNA decay in upper layer in May, therefore no significant differences in MGC between individual layers were observed. “

Line 532: "Pacyfic" should be spelled Pacific- has been corrected

## 2. **Responses to the Editor**

When submitting your revision, we need you to address these additional requirements.

1. Please ensure that your manuscript meets PLOS ONE's style requirements, including those for file naming. The PLOS ONE style templates can be found at 
<https://journals.plos.org/plosone/s/file?id=wjVg/PLOSOne_formatting_sample_main_body.pdf> and 
<https://journals.plos.org/plosone/s/file?id=ba62/PLOSOne_formatting_sample_title_authors_affiliations.pdf>

The manuscript has been edited according to the PLOSOne recommendations

2. In your Methods section, please include a comment about the state of the fish following this research. Were they euthanized or housed for use in further research? If any animals were sacrificed by the authors, please include the method of euthanasia and describe any efforts that were undertaken to reduce animal suffering.

We have added a following sentence to the Methods section: ” Fish were housed for use in further research and animals were not sacrificed by the authors.”

3. We note that you are reporting an analysis of a microarray, next-generation sequencing, or deep sequencing data set. PLOS requires that authors comply with field-specific standards for preparation, recording, and deposition of data in repositories appropriate to their field. Please upload these data to a stable, public repository (such as ArrayExpress, Gene Expression Omnibus (GEO), DNA Data Bank of Japan (DDBJ), NCBI GenBank, NCBI Sequence Read Archive, or EMBL Nucleotide Sequence Database (ENA)). In your revised cover letter, please provide the relevant accession numbers that may be used to access these data. For a full list of recommended repositories, see <http://journals.plos.org/plosone/s/data-availability#loc-omics> or <http://journals.plos.org/plosone/s/data-availability#loc-sequencing>.

The result of amplicon high throughput sequencing leading to demonstration of SL2 assay specificity has been submitted to NCBI Sequence Read Archive. The number of submissions has been added to the manuscript (BioSample accession SAMN29936430)

4. Please update your submission to use the PLOS LaTeX template. The template and more information on our requirements for LaTeX submissions can be found at <http://journals.plos.org/plosone/s/latex>.

The manuscript has been edited according to LaTeX template.

5. In your Data Availability statement, you have not specified where the minimal data set underlying the results described in your manuscript can be found. PLOS defines a study's minimal data set as the underlying data used to reach the conclusions drawn in the manuscript and any additional data required to replicate the reported study findings in their entirety. All PLOS journals require that the minimal data set be made fully available. For more information about our data policy, please see <http://journals.plos.org/plosone/s/data-availability>.

Upon re-submitting your revised manuscript, please upload your study’s minimal underlying data set as either Supporting Information files or to a stable, public repository and include the relevant URLs, DOIs, or accession numbers within your revised cover letter. For a list of acceptable repositories, please see <http://journals.plos.org/plosone/s/data-availability#loc-recommended-repositories>. Any potentially identifying patient information must be fully anonymized.

All important information regarding Data Availability can be found in the cover letter.

We will update your Data Availability statement to reflect the information you provide in your cover letter.

6. We note that you have stated that you will provide repository information for your data at acceptance. Should your manuscript be accepted for publication, we will hold it until you provide the relevant accession numbers or DOIs necessary to access your data. If you wish to make changes to your Data Availability statement, please describe these changes in your cover letter and we will update your Data Availability statement to reflect the information you provide.

We have provided information about DOI number in the cover letter.

7. Please amend either the title on the online submission form (via Edit Submission) or the title in the manuscript so that they are identical.

Thank you for this insight. We have made the titles identical.

8. Please review your reference list to ensure that it is complete and correct. If you have cited papers that have been retracted, please include the rationale for doing so in the manuscript text, or remove these references and replace them with relevant current references. Any changes to the reference list should be mentioned in the rebuttal letter that accompanies your revised manuscript. If you need to cite a retracted article, indicate the article’s retracted status in the References list and also include a citation and full reference for the retraction notice.

The following citation was added to the main text: *Harrison JB, Sunday JM, Rogers SM. Predicting the fate of eDNA in the environment and implications for studying biodiversity. Proceedings of the Royal Society B: Biological Sciences. 2019;286(1915):20191409.*

While revising your submission, please upload your figure files to the Preflight Analysis and Conversion Engine (PACE) digital diagnostic tool, <https://pacev2.apexcovantage.com/>. PACE helps ensure that figures meet PLOS requirements. To use PACE, you must first register as a user. Registration is free. Then, login and navigate to the UPLOAD tab, where you will find detailed instructions on how to use the tool. If you encounter any issues or have any questions when using PACE, please email PLOS at [figures@plos.org](mailto:figures@plos.org). Please note that Supporting Information files do not need this step.

This has been done
